# Molecular and Cellular Effects of C-peptide—New Perspectives on an Old Peptide

**DOI:** 10.1080/15438600490424479

**Published:** 2004

**Authors:** John Wahren, Jawed Shafqat, Jan Johansson, Alexander Chibalin, Karin Ekberg, Hans Jörnvall

**Affiliations:** 1 Department of Surgical Sciences Section of Clinical Physiology and Department of Medical Biochemistry and Biophysics Karolinska Institutet Stockholm Sweden; 2 Creative Peptides AB, P.O. Box 6753 Stockholm SE-113 85 Sweden

## Abstract

New results present C-peptide as a biologically active peptide hormone in its own right. Although C-peptide is formed from proinsulin and cosecreted with insulin, it is a separate entity with biochemical and physiological characteristics that differ from those of insulin. There is direct evidence of stereospecific binding of C-peptide to a cell surface receptor, which is different from those for insulin and other related hormones. The C-peptide binding site is most likely a G–protein–coupled receptor. The association constant for C-peptide binding is approximately 3 × 10^9^M^-1^. Saturation of the binding occurs already at a concentration of about 1 nM, which explains why C-peptide effects are not observed in healthy subjects. Binding of C-peptide results in activation of Ca^2+^ and MAPK-dependent pathways and stimulation of Na^+^,K^+^-ATPase and eNOS activities. The latter 2 enzymes are both deficient in several tissues in type 1 diabetes. There is some evidence that C-peptide, and insulin may interact synergistically on the insulin signaling pathway. Clinical evidence suggests that replacement of C-peptide, together with regular insulin therapy, may be beneficial in patients with type 1 diabetes and serve to retard or prevent the development of long-term complications.


*Experimental Diab. Res.*, 5:15–23, 2004
Copyright © Taylor and Francis Inc.
ISSN: 1543-8600 print / 1543-8619 online
DOI: 10.1080/15438600490424479

Taylor & Francis
Taylor & Francis Group

### Molecular and Cellular Effects of C-peptide—New Perspectives on an Old Peptide

**John Wahren, Jawed Shafqat, Jan Johansson, Alexander Chibalin, Karin Ekberg, and Hans Jörnvall**

*Department of Surgical Sciences, Section of Clinical Physiology, and Department of Medical Biochemistry and Biophysics, Karolinska Institutet, Stockholm, Sweden*

**New results present C-peptide as a biologically active peptide hormone in its own right. Although C-peptide is formed from proinsulin and cosecreted with insulin, it is a separate entity with biochemical and physiological characteristics that differ from those of insulin. There is direct evidence of stereospecific binding of C-peptide to a cell surface receptor, which is different from those for insulin and other related hormones. The C-peptide binding site is most likely a G–protein–coupled receptor. The association constant for C-peptide binding is approximately $3 \times 10^9$ M$^{-1}$. Saturation of the binding occurs already at a concentration of about 1 nM, which explains why C-peptide effects are not observed in healthy subjects. Binding of C-peptide results in activation of $Ca^{2+}$ and MAPK-dependent pathways and stimulation of $Na^+$,$K^+$-ATPase and eNOS activities. The latter 2 enzymes are both deficient in several tissues in type 1 diabetes. There is some evidence that C-peptide, and insulin may interact synergistically on the insulin signaling pathway. Clinical evidence suggests that replacement of C-peptide, together with regular insulin therapy, may be beneficial in patients with type 1 diabetes and serve to retard or prevent the development of long-term complications.**

**Keywords** Endothelial Nitric Oxide Synthase; G Protein Coupled Receptor; Insulinomimetic Effects; Intracellular $Ca^{2+}$; Membrane Binding; Mitogen-Activated Protein Kinase; $Na^+$,$K^+$ ATPase; Proinsulin; Regional Blood Flow

Received 23 January 2003; accepted 2 April 2003.
Address correspondence to Professor John Wahren, Creative Peptides Sweden AB, P.O. Box 6753, SE-113 85 Stockholm, Sweden. E-mail: john.wahren@creativepeptides.se

### INTRODUCTION

Several experimental and clinical studies published in the past decade have consistently demonstrated that proinsulin C-peptide is not as biologically inert as was previously thought. Thus, C-peptide binds specifically to cell membranes and activates intracellular signaling pathways, resulting in stimulation of both $Na^+$,$K^+$-ATPase and endothelial nitric oxide synthase (eNOS) activities (Johansson et al., 2002b; Wahren et al., 2000). Studies in C-peptide deficient type 1 diabetes patients have shown that replacement of C-peptide is accompanied by significant improvements in renal and nerve function. Thus, C-peptide replacement partially corrects both the glomerular hyperfiltration and the microalbuminuria that characterize the early stages of diabetic nephropathy (Johansson et al., 1992b, 2000). Likewise, sensory nerve conduction velocity, vibration thresholds, and autonomic nerve function, which are altered early on in the course of diabetic neuropathy, improve significantly after C-peptide replacement therapy in type 1 diabetes patients (Ekberg et al., 2003; Johansson et al., 1996, 2000). Data from animal studies suggest that the amelioration of nerve dysfunction may be related to a C-peptide–induced increase in nerve blood flow (Cotter et al., 2003) and/or to augmented endoneural $Na^+$,$K^+$-ATPase activity (Ido et al., 1997; Sima et al., 2001). These biochemical and clinical findings have prompted the notion that C-peptide deficiency could contribute to the development of long-term complications in type 1 diabetes, and that C-peptide administration, together with insulin therapy, may be beneficial in the treatment and prevention of such complications. In this report, we review the recently presented molecular and cellular effects of C-peptide.

### C-PEPTIDE STRUCTURE AND MOLECULAR PROPERTIES

C-peptide originates from the mid portion of proinsulin, corresponding to the segment between the insulin A and B chains. It is a highly acidic peptide, with 5 acidic residues in the human form and up to 7 in other species, with no counterbalance from basic residues. The human form is a 31-residue peptide. The interspecies variability is considerable, with regard to both the amino acid sequence and the exact number of residues; it is in the central region of the molecule that the size varies. Some species, including the mouse, have 2 isoforms that also differ quite markedly (5 positions between the 2 mouse C-peptide forms; Figure 1).

The extent of sequence variability in C-peptide resembles that of relaxin, another hormone from the same protein family, but is greater than that of many other bioactive peptides (Table 1). In mammals, however, 8 residues in C-peptide are quite well conserved. They correspond to the following positions in the human C-peptide: 1 (Glu), 3 (Glu), 6 (Gln), 11 (Glu), 12 (Leu), 26 (Leu), 27 (Glu), and 31 (Gln). From this type of partial structural conservation, it is obvious that acidic residues (especially Glu) are important for C-peptide function, not only overall (the acidic nature of the whole peptide), but also at specific positions (conserved, or nearly so). One of these residues, Glu27, has also been ascribed a particular role in the light of direct measurements of cellular responses with different analogues (Pramanik et al., 2001; Shafqat et al., 2002; Table 2). Notably, free Glu also exhibits some C-peptide–like activity in vitro and can partly displace cell-bound C-peptide (Ohtomo et al., 1998; Pramanik et al., 2001).

C-peptide does not adopt a defined conformation, it assumes a "random coil" structure in solution (Henriksson et al., 2000). Trifluoroethanol may induce an N-terminally positioned helical structure (Henriksson et al., 2000), but in this solvent, helical formation is frequent also for other peptides. Although particular structural elements have been suggested to be important in membrane interactions of C-peptide (Ido et al., 1997), no direct evidence has been presented for the presence

| | |
|---|---|
| Human | EAEDLQVGQVELGGGPGAGSLQPLALEGSLQ |
| Mouse 1 | EVEDPQVEQLELGGSP - - GDLQTLALEVARQ |
| Mouse 2 | EVEDPQVAQLELGGGPGAGDLQTLALEVAQQ |

**FIGURE 1**
Primary structures of human and mouse C-peptides. Underlined residue symbols indicate species differences. As shown, the mouse has 2 C-peptide isoforms, differing in internal size (gap positions).

**TABLE 1**
Sequence variability of peptide hormones relating to pancreatic functions and including all the insulin-family members

| | Comparison of human and mouse forms of bioactive peptides | |
|---|---|---|
| Hormone | Variability (%) | Number of variable versus total residues |
| Somatostatin | 0 | 0 of 14 |
| VIP | 0 | 0 of 28 |
| Glucagon | 0 | 0 of 29 |
| IGF-1 | 6 | 4 of 70 |
| GIP | 7 | 3 of 42 |
| Insulin | 8 | 4 of 51 |
| CCK | 9 | 3 of 33 |
| IGF-2 | 9 | 6 of 67 |
| Secretin | 15 | 4 of 27 |
| Pancreatic polypeptide | 17 | 6 of 36 |
| GRP | 19 | 5 of 27 |
| C-peptide* | 35 | 11 of 31 |
| Relaxin | 56 | 30 of 54 |

*Note*. Structures are from data banks (Swiss Prot) and completed genome sequences (Ensemble).

*Data for C-peptide are based on comparison with the mouse type 1 C-peptide.

**TABLE 2**
Correlation of results with C-peptide, the C-terminal pentapeptide fragment, and its Ala-substituted analogues, using 2 different methods, fluorescence correlation spectroscopy (FCS) and increase in intracellular calcium concentration ($[Ca^{2+}]_i$)

| Peptides | FCS | $[Ca^{2+}]_i$ |
|---|---|---|
| Full length C-peptide | +++ | +++ |
| C-terminal pentapeptide EGSLQ | +++ | +++ |
| EGSLQ analogues | | |
| AGSLQ | − | − |
| EASLQ | +++ | + |
| EGALQ | ++ | +++ |
| EGSAQ | + | + |
| EGSLA | ++ | ++ |

*Note*. The plus signs indicate relative responses in the 2 assay methods. As shown, the general agreement is excellent, with deviations for only some of the analogue forms.

Data from Rigler et al. (1999); Pramanik et al. (2001); Shafqat et al. (2002).

of membrane-induced conformational changes for C-peptide (Henriksson et al., 2000).

The pronounced interspecies structural variability of C-peptide has been difficult to interpret in functional terms. Peptides with defined functions are often well conserved (Table 1) and the absence of strict conservation in C-peptide probably contributed to the long delay before C-peptide was recognized as a peptide hormone. The apparent discrepancy between defined functions and a low structural conservation may indicate either that only limited regions are important for C-peptide function (thus limiting the extent of necessary conservation), or that essentially only charge interactions are important (limiting exact sequence conservation as long as at least some Glu residues are conserved), or that a conserved function exists only in mammals (where C-peptide conservation is reasonable) rather than within the whole vertebrate line. Significantly, and in support of these interpretations, the short C-terminal pentapeptide fragment of C-peptide has been shown to be sufficient for C-peptide responses in assays (*cf* below) using $Na^+$,$K^+$-ATPase (Ohtomo et al., 1998), fluorescence correlation spectroscopy (Rigler et al., 1999), and intracellular calcium concentrations (Shafqat et al., 2002).

In conclusion, the structural and evolutionary properties of C-peptide show that it lacks a stable conformation in physiological solutions and has a high degree of structural variability. Moreover, C-peptide has an important C-terminal segment and elicits measurable molecular and cellular effects that correlate with functional differences among analogues in a manner compatible with hormonal function. In addition, C-peptide is of structural importance, recognized long ago, in the folding of the parent proinsulin molecule.

### MEMBRANE INTERACTIONS AND CELLULAR EFFECTS

### Binding of C-peptide to Cell Membranes

Specific binding of C-peptide to cell membranes has been studied with a new technique, fluorescence correlation spectroscopy (FCS). In FCS, the Brownian movements of a fluorophore-marked ligand (tetramethylrhodamine-labeled C-peptide) are recorded after excitation by a sharply focused laser beam. The measurements are performed in a small volume element ($<1 \times 10^{-15}$ L) that can be shifted from the incubation medium above a cell growing in culture to the cell membrane. The autocorrelation function that describes the spontaneous fluctuations in fluorescence intensity within the volume element is calculated, making it possible to determine the diffusion time of the labeled peptide—when free in the incubation medium or bound to the cell membrane—and to characterize the nature of the peptide-cell surface interaction. The FCS technique offers higher detection sensitivity and an improved signal-to-noise ratio as compared to the conventional radioligand-binding method (Eigen and Rigler, 1994; Maiti et al., 1997).

Using the FCS technique with rhodamine-labeled C-peptide, C-peptide has been shown to bind specifically to renal convoluted tubular cells, skin fibroblasts, and saphenous vein endothelial cells (Rigler et al., 1999). All cell types were human and homologous C-peptide was used. The specificity of the binding was evidenced by displacement of bound C-peptide after addition of excess unlabeled C-peptide. Addition to the incubation medium of scrambled C-peptide (a 31–amino acid residue peptide with its components arranged in random order) or of the enantio (D-form) C-peptide failed to displace the bound C-peptide, findings which attest to the specificity and chiral nature of the C-peptide binding to the cell membrane. Incubation with insulin, proinsulin, insulin-like growth factor (IGF)-I, IGF-II, or neuropeptide Y (NPY) does not result in competitive displacement of bound C-peptide, demonstrating that there is no cross-reactivity between these hormones and their receptors. Likewise, C-peptide in supraphysiological concentrations did not displace bound insulin. Preincubation of the cells with pertussis toxin abolished the C-peptide binding to cell membranes (Rigler et al., 1999). The latter finding is consistent with an allosteric mechanism being involved in C-peptide signal transduction. Prior to exposure to pertussis toxin, there was a high-affinity interaction between C-peptide and its receptor, reflecting one configuration of the receptor; after pertussis toxin exposure, only a small component of low-affinity interaction remained, suggesting a new configuration of the receptor, secondary to an effect of pertussis toxin on the $\alpha$-subunit of the G-protein (Hamm, 1998).

The binding curve and the Scatchard diagram describing the interaction between C-peptide and renal tubular cells are illustrated in Figure 2. The estimated association constant is approximately $3 \times 10^9$ $M^{-1}$, assuming one receptor binding site per C-peptide molecule. Similar or slightly lower association constants were found for C-peptide binding to fibroblasts and endothelial cells (Rigler et al., 1999). The C-peptide binding curve indicates that saturation of binding occurs already at a concentration of approximately 0.9 nM (Figure 2). This finding may be of clinical importance, because no effects of C-peptide can be demonstrated in healthy individuals or animals (Hoogwerf et al., 1986; Johansson et al., 1992a; Sima et al., 2001; Wójcikowski et al., 1983). It is only in C-peptide–negative type 1 diabetes patients or animals that several physiological effects of C-peptide have been found (*cf.* Wahren et al., 2000). This seeming discordance can now be explained by the finding that even a low concentration of C-peptide leads to saturation of C-peptide binding sites. In healthy subjects or animals, receptor saturation probably occurs already at the ambient

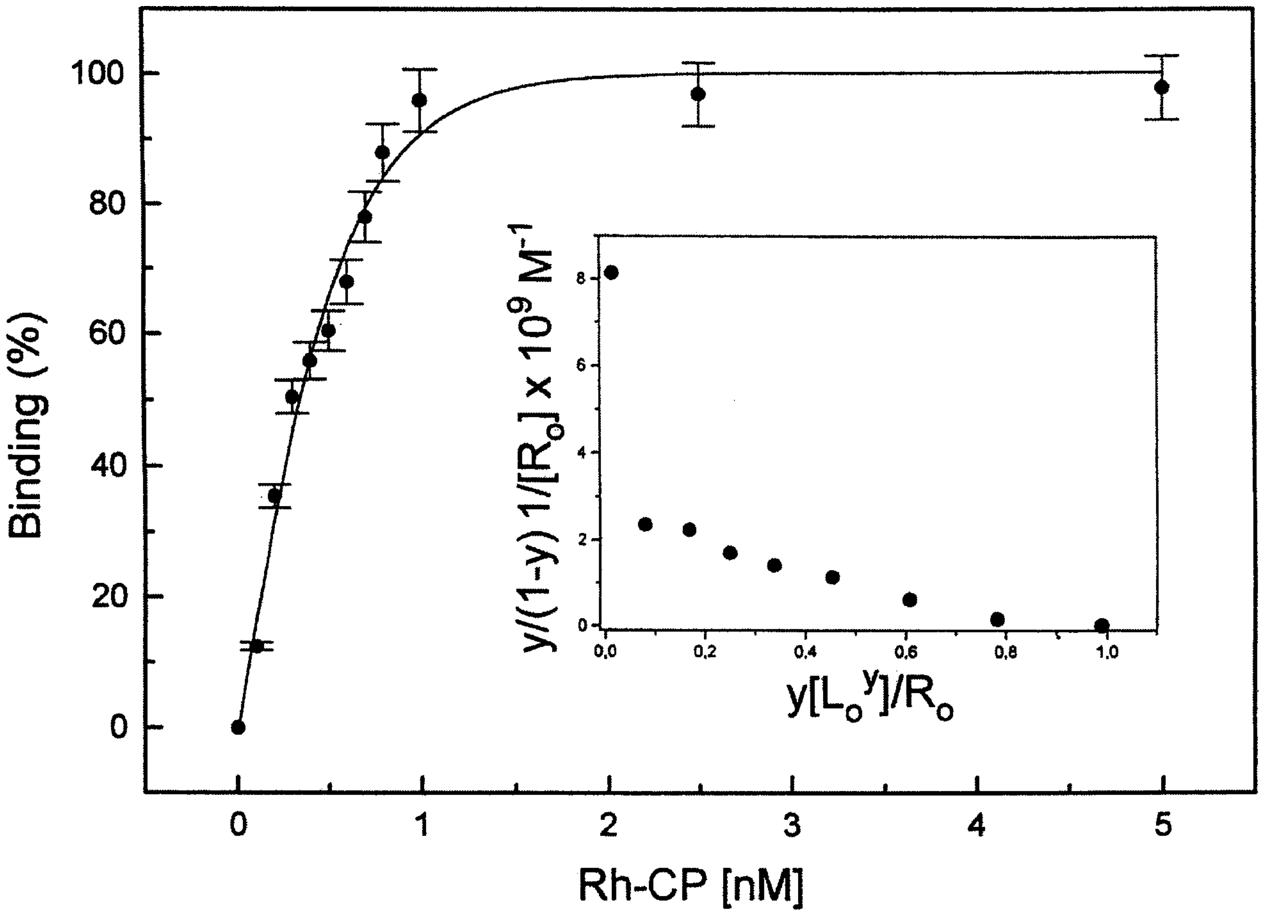

**FIGURE 2**

Binding of rhodamine-labeled C-peptide to cell membranes of renal tubular cells. Fractional saturation (y) of the membrane-bound labeled ligand is presented as a function of the C-peptide concentration (L) in the binding medium. The binding curve corresponds to $K_{ass} = 3.3 \times 10^9\ M^{-1}$. A corresponding Scatchard plot is shown in the insert. Reproduced from (Rigler et al., 1999, copyright [1999] National Academy of Sciences, U. S. A.).

C-peptide concentration and no further physiological effects are to be expected from an increased concentration.

Species specificity with regard to C-peptide binding has not been studied in detail but human C-peptide, albeit at supraphysiological concentrations, is reported to improve nerve function and lower both glomerular hyperfiltration and urinary albumin excretion in the diabetic rat (Huang et al., 2002; Ido et al., 1997). Likewise, a C-peptide bioassay based on $Ca^{2+}$ release (see below) shows cross-reactivity and effects of both the rat C-peptide and the rat C-terminal pentapeptide in human cells (Shafqat et al., 2002).

The radioligand method for hormonal binding studies has also been employed for the evaluation of C-peptide cell membrane interactions. Tyrosylated $^{125}$I-labeled homologous C-peptide has been reported to bind specifically to cultured rat islet cells (Flatt et al., 1986). However, subsequent attempts to document specific binding of radiolabeled C-peptide to crude cell membranes from skeletal muscle (Zierath et al., 1996) and to human renal tubular cells (Rigler et al., 1999) were not successful. Neither could binding to human lymphoblasts be demonstrated (Jehle et al., 1996), but bound labeled proinsulin can be displaced by C-peptide (Jehle et al., 1996, 1999). The variable results and the difficulties in demonstrating cellular binding of C-peptide using radioligand techniques have not been fully explained but the relative paucity of C-peptide binding sites per unit cell surface area (Flatt et al., 1986; Rigler et al., 1999) may contribute. The structure of the C-peptide receptor is not known, but it has been found that treatment of cells with detergents causes release of solubilized macromolecules that bind specifically to C-peptide (Henriksson et al., 2001).

Bioactive peptides generally exert their cellular effects via specific binding of a limited region of the ligand molecule to the receptor. It has been shown that the C-terminal pentapeptide of human C-peptide (EGSLQ) can competitively displace the bound, full-length peptide (Rigler et al., 1999), indicating the involvement of the C-terminal segment as an "active site" in the binding process. Studies with sequential alanine substitution of the pentapeptide demonstrated that the pentapeptide N-terminal glutamic acid residue (Glu27) is of particular importance for the binding process (Pramanik et al., 2001) and for eliciting an intracellular $Ca^{2+}$ response (Shafqat et al., 2002; Table 2).

In summary, direct binding studies demonstrate specific binding of C-peptide to cell surfaces and to solubilized cell components. Association constants, binding curves, and displacement characteristics have been documented at physiologically relevant concentrations. The C-terminal pentapeptide has been shown to be capable of competitively displacing the bound full-length peptide, indicating that this segment is involved in the binding process. A functional receptor has still to be identified, but the available data on the binding of C-peptide to cell membranes provide a molecular basis for its further effects, discussed below.

### Cellular Effects of C-peptide

*Intracellular Calcium Concentrations*

Several lines of evidence suggest that intracellular $Ca^{2+}$ functions as a second messenger in the C-peptide signal transduction. Exposure of rat convoluted tubular cells in primary culture to homologous C-peptide in the physiological concentration range is accompanied by a prompt elevation of the intracellular $Ca^{2+}$ concentration (Ohtomo et al., 1996). The human full-length C-peptide and its C-terminal pentapeptide are equally effective in increasing the intracellular $Ca^{2+}$ in human renal cells, but scrambled C-peptide has no effect, confirming the specificity of the signal (Shafqat et al., 2002). A pentapeptide analogue with Glu1 replaced by Ala fails to increase intracellular $Ca^{2+}$, in agreement with the importance of this residue for the binding process (Pramanik et al., 2001). Addition of a calcium chelator to the medium abolishes the effect of C-peptide, suggesting that the effect is elicited via an influx of extracellular $Ca^{2+}$ rather than by release from intracellular depots (Wallerath et al., 2003; Ohtomo et al., 1996). Addition of pertussis toxin to the medium also prevents the effects of both C-peptide and the pentapeptide segment on intracellular $Ca^{2+}$ concentrations, again indicating G-protein involvement in the C-peptide signaling pathway (Shafqat et al., 2002).

*Mitogen-Activated Protein Kinase (MAPK)*

C-peptide is reported to induce phosphorylation of the MAPK extracellular signal-regulated kinases (ERK 1/2) of a fibroblast cell line (Swiss 3T3), whereas reverse-sequence C-peptide and an all-D-amino acid C-peptide failed to do so (Kitamura et al., 2001). Moreover, phosphorylation of ERK 1/2 is also observed in human renal tubular cells upon incubation with homologous C-peptide and its C-terminal pentapeptide segment (Chibalin et al., 2002). Preincubation of the cells with pertussis toxin or with PD98059, a MAPK kinase 1 inhibitor, abolishes the stimulatory effect on ERK phosphorylation. C-peptide has also been found to stimulate both p38 stress-activated protein kinase (p38 MAPK) and ERK 1/2 activity and phosphorylation in mouse lung capillary endothelial cells (Kitamura et al., 2002). In addition, C-peptide stimulation induces phosphorylation and DNA binding of transcription factors and these effects are blocked by a p38 MAPK inhibitor but not by an ERK 1/2 inhibitor (Kitamura et al., 2002). Altogether, these data constitute evidence that MAPK phosphorylation is involved in C-peptide signal transduction in a several cell types.

*$Na^{+}$,$K^{+}$-ATPase*

The level of $Na^{+}$,$K^{+}$-ATPase activity is reduced in several tissues in experimental and human diabetes (De La Tour et al., 1998; Ido et al., 1997). C-peptide is reported to exert a stimulatory effect on $Na^{+}$,$K^{+}$-ATPase activity under both in vitro and in vivo conditions. Exposure of rat convoluted tubular segments to homologous C-peptide and its fragments results in an increase in $Na^{+}$,$K^{+}$-ATPase activity as measured by hydrolysis of [$^{32}$P]ATP (Ohtomo et al., 1996, 1998; Tsimaratos et al., 2002). In rat tubular collecting duct cells, stimulation of $Na^{+}$,$K^{+}$-ATPase activity is reported to be mediated via protein kinase $C_{\alpha}$ (Tsimaratos et al., 2003). Similarly, studies using human tubular cells in primary culture and ouabain-sensitive uptake of $^{86}Rb^{+}$ as a marker of $Na^{+}$,$K^{+}$-ATPase activity have confirmed a concentration-dependent effect of human C-peptide on $Na^{+}$,$K^{+}$-ATPase activity at physiological concentrations (Chibalin et al., 2002). The stimulatory effect of C-peptide on $Na^{+}$,$K^{+}$-ATPase in human renal cells was abolished both by a protein kinase C inhibitor and by PD 98059, and the data indicate that regulatory properties and phosphorylation sites for the human enzyme differ from those of the rodent enzyme. Interestingly, C-peptide's ability to stimulate $Na^{+}$,$K^{+}$-ATPase activity is augmented by more than 2 orders of magnitude in the presence of subthreshold concentrations of NPY (Ohtomo et al., 1996). NPY is known to elicit phosphorylation of ERK 1/2 (Mullins et al., 2002), suggesting that the interaction between the 2 peptides may occur at the MAPK level. Pretreatment of tubular segments or cells with pertussis toxin or incubation of the cells in a $Ca^{2+}$-free medium blocks the C-peptide effect (Chibalin et al., 2002; Ohtomo et al., 1996). Likewise, the stimulatory effect on $Na^{+}$,$K^{+}$-ATPase is abolished by pretreatment with FK 506, a specific inhibitor of the $Ca^{2+}$-calmodulin–dependent protein phosphatase 2B (Ohtomo et al., 1996). The C-terminal pentapeptide segment has been found to be as potent as the full-length C-peptide in stimulating

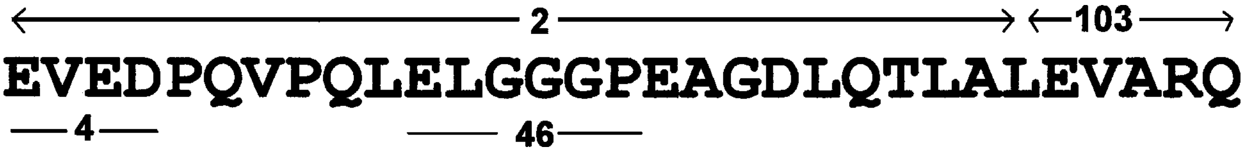

**FIGURE 3**

Effects of selected rat C-peptide fragments on $Na^+$,$K^+$-ATPase activity in rat proximal convoluted tubular segments. The numbers indicate the percent activity of each fragment in relation to that of the intact C-peptide. Data from Ohtomo et al. (1998).

$Na^+$,$K^+$-ATPase activity (Figure 3) (Chibalin et al., 2002; Ohtomo et al., 1998). In contrast, effects of the truncated des(27–31) C-peptide or an N-terminal segment are insignificant, whereas some stimulatory effect is observed for middle segments (Ohtomo et al., 1998) (Figure 3). Replacement of C-peptide in type 1 diabetic rats for periods of 2 to 8 months results in partial restoration of sciatic nerve $Na^+$,$K^+$-ATPase activity (Ido et al., 1997; Sima et al., 2001). Moreover, red blood cell $Na^+$,$K^+$-ATPase activity is reduced in patients with type 1 diabetes (Finotti and Palatini, 1986; McMillan et al., 1978). The reduction is proportional to the simultaneous decrease in C-peptide plasma concentration (Dufayet De La Tour et al., 1998) and can be corrected by C-peptide administration (Forst et al., 2000). Thus, the available evidence supports the existence of a direct relationship between C-peptide levels and $Na^+$,$K^+$-ATPase activities in renal and nerve tissues under both in vitro and in vivo enconditions.

*Endothelial Nitric Oxide Synthase Stimulation*

The long-term complications accompanying type 1 diabetes are primarily attributed to microvascular dysfunction. C-peptide is reported to stimulate blood flow in several tissues (Cotter et al., 2003; Ekberg et al., 2001; Hansen et al., 2002; Johansson et al., 1992a, 1992b, 2004) in patients with type 1 diabetes and in animals with experimental diabetes. The increase in blood flow is accompanied by reduced vascular resistance and increased capillary recruitment (Lindström et al., 1996). In type 1 diabetes patients, C-peptide stimulates forearm blood flow in a concentration-dependent manner (Ekberg et al., 2001) and the vasodilatory effect of C-peptide on forearm blood flow is completely abolished when C-peptide is administered into the brachial artery together with an NOS inhibitor (Cotter et al., 2003; Johansson et al., 1999). Likewise, in streptozotocin-induced diabetic rats, C-peptide stimulates nerve blood flow and this effect is completely abolished when C-peptide is coadministered with an NO blocker (Cotter et al., 2003).

There is in vitro evidence for the notion that C-peptide elicits vasodilatation via release of NO. Using a reporter cell assay and bovine aortic endothelial cells, C-peptide was found to increase the endothelial release of NO in a concentration-dependent and time-dependent manner (Wallerath et al., 2003). This effect, measured as cGMP formation in the reporter cells (RFL-6 fibroblasts) occurred within 2 minutes. The effect was completely abolished in the presence of an NOS inhibitor, and no effect was found when a calcium binding agent was added to the incubation medium (Wallerath et al., 2003), suggesting that the vasodilatory effect of C-peptide is $Ca^{2+}$-dependent and direct. Although increased expression of eNOS mRNA levels in lung tissue has been reported for rats injected with C-peptide (Scalia et al., 2000), no such effect was apparent in the bovine aortic endothelial cells (Wallerath et al., 2003). C-peptide may also exert other NO-mediated effects; a cardioprotective effect against leukocyte-mediated reperfusion injury has been reported in the isolated rat heart (Young et al., 2000), and C-peptide is a potent inhibitor of leucocyte-endothelium interactions. This effect is specifically related to inhibition of endothelial cell adhesion molecules via stimulation of NO release from the vascular endothelium (Scalia et al., 2000).

*Insulinomimetic Effects of C-peptide*

C-peptide at physiological concentrations has been found to mimic insulin effects in L6 myoblasts; it activates insulin receptor tyrosine kinase, insulin receptor substrate (IRS1) phosphorylation, phosphoinositol 3-kinase (PI3K) activity, and MAPK phosphorylation, besides augmenting glycogen synthesis and increasing amino acid uptake (Grunberger et al., 2001). However, other studies indicate that C-peptide stimulates glucose transport in human muscle cells without involving the insulin receptor or causing tyrosine kinase or PI3K activation (Zierath et al., 1996). C-peptide at up to supraphysiological concentrations fails to stimulate glycogen synthesis in mouse soleus muscle (Shashkin et al., 1997). In vivo studies in diabetic rats demonstrated a marked stimulation of whole body glucose turnover in the short term (Ling et al., 1999). Similarly, a mild to modest stimulation of glucose turnover was seen in type 1 diabetes patients in short-term studies (Johansson et al., 1992b), but little or no effect on blood glucose levels or metabolic control has been observed in long-term (3 months) studies (Ekberg et al., 2003; Johansson et al., 2000).

Additional receptor systems, not involving G-proteins, have also been associated with C-peptide. Interactions between

C-peptide and receptors with catalytic activity are suggested by results showing that C-peptide may alter the activity of protein tyrosine phosphatase (Li et al., 2001), an enzyme that may inactivate the insulin signaling pathway by dephosphorylation of the insulin receptor, IRS1, and MAPK. C-peptide interactions with ligand-gated ion channels coupled to glutamate receptors have also been considered both in view of the capacity of free glutamic acid to partly displace C-peptide from cell membranes, and considering the importance of N-terminal Glu for binding and biological activity of intact C-peptide and its C-terminal pentapeptide (Bourguignon et al., 1994; Pramanik et al., 2001).

*Nonreceptor Interactions*

It has been suggested that the effects of C-peptide may be mediated by a direct interference with the structure of the cell membrane instead of by binding to a stereospecific receptor (Ido et al., 1997). Human C-peptide in supraphysiological concentrations, an all D-amino acid C-peptide and a retrosequence C-peptide were all found to partially correct diabetes-induced vascular dysfunction in a rat model of type 1 diabetes (Ido et al., 1997). The findings were taken to indicate that the C-peptide effect may have been mediated in a manner similar to that for antibiotic peptides, involving clustering on the cell membrane and pore or ion channel formation (Bessalle et al., 1990). However, direct measurements indicate that C-peptide fails to associate in a stable manner with lipid-containing membranes or micelles (Henriksson et al., 2000). Moreover, it should be noted that C-peptide does not possess the characteristics of a channel-forming peptide; it does not show a tendency to self-associate, is overall hydrophilic and polar (negatively charged), and lacks a specific structure in solution (Henriksson et al., 2000). It is therefore unlikely that the effects of C-peptide are mediated via conformation-dependent, nonchiral membrane interactions.

The currently available information presented above provides a general outline of the intracellular signal transduction pattern for C-peptide (Figure 4). This pattern involves

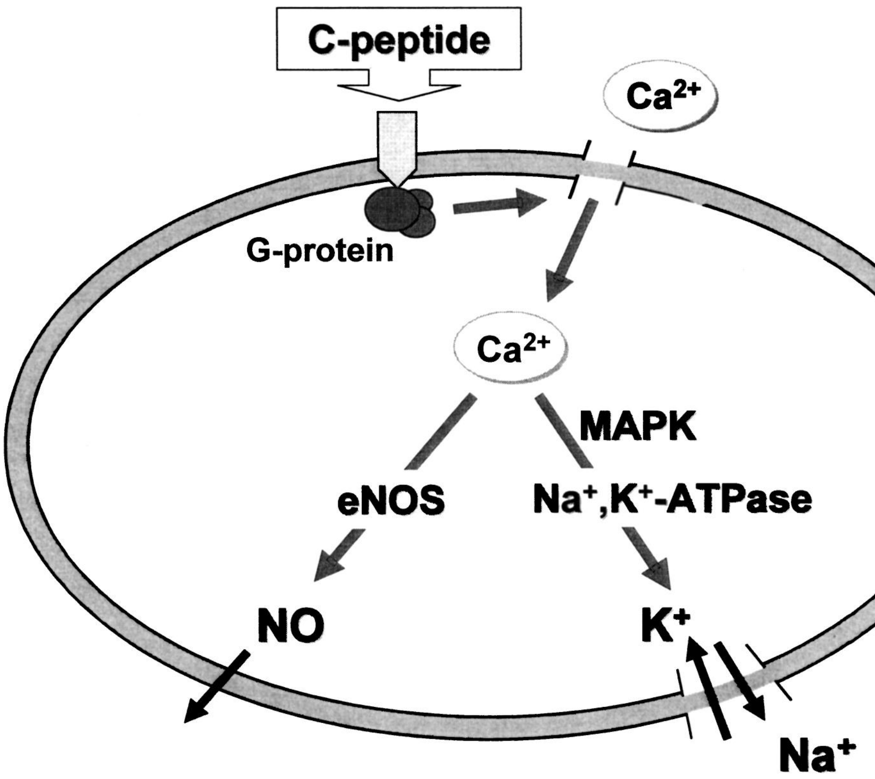

**FIGURE 4**

Schematic representation of cellular effects of C-peptide. The available evidence is compatible with the hypothesis that C-peptide binds to a cell membrane receptor coupled to a pertussis toxin sensitive G-protein. The G-protein signal opens $Ca^{2+}$ channels, resulting in a rise in the intracellular $Ca^{2+}$ concentration, with subsequent stimulation of endothelial nitric oxide synthase (eNOS) activity, formation of NO, and vasodilatation. There is also stimulation of the MAP kinase signaling pathway, resulting in augmented $Na^+$,$K^+$-ATPase activity. In addition, C-peptide and insulin may interact synergistically on the insulin signaling pathway.

interaction of the peptide with a G-protein–coupled membrane receptor and activation of $Ca^{2+}$-dependent intracellular signaling pathways. The latter include phosphorylation of protein kinase C and the MAPK system, eliciting increased activity of $Na^+$,$K^+$-ATPase. The increase in the intracellular $Ca^{2+}$ concentration also gives rise to increased eNOS activity, NO formation, and vasodilation. In addition, some studies indicate that C-peptide and insulin may interact synergistically on the insulin signaling pathway.

### REFERENCES

Bessalle, R., Kapitkovsky, A., Gorea, A., Shalit, I., and Fridkin, M. (1990) All-D-magainin: Chirality, antimicrobial activity and proteolytic resistance. *FEBS Lett.*, **274,** 151–155.

Bourguignon, J., Alvarez Gonzalez, M., Gerard, A., and Franchimont, P. (1994) Gonadotropin relasing hormone inhibitory autofeed back by subproducts antagonist at *N*-methyl-D-aspartate receptors: A model of autocrine regulation of peptide secretion. *Endocrinology,* **134,** 1589–1592.

Chibalin, A., Dumitresco, A., Ehrén, I., Ekberg, K., and Wahren, J. (2002) C-peptide stimulates Na,K-ATPase activity via MAP-kinase dependent pathway in human renal tubular cells. *Diabetologia,* **45(suppl 2**), A350.

Cotter, M., Ekberg, K., Wahren, J., and Cameron, N. (2003) Effects of proinsulin C-peptide in experimental diabetic neuropathy: Vascular actions and modulation by nitric oxide synthase inhibition. *Diabetes,* **52,** 1812–1817.

Dufayet De La Tour, D., Raccah, D., Jannot, M., Coste, T., Rougerie, C., and Vague, P. (1998) Erythrocyte Na/K ATPase activity and diabetes: Relationship with C-peptide level. *Diabetologia,* **41,** 1080–1084.

Eigen, M., and Rigler, R. (1994) Sorting single molecules: Application to diagnostics and evolutionary biotechnology. *Proc. Natl. Acad. Sci. U. S. A.,* **91,** 5740–5747.

Ekberg, K., Brismar, T., Johansson, B.-L., Jonsson, B., Lindström, P., and Wahren, J. (2003) Amelioration of sensory nerve dysfunction by C-peptide in patients with type 1 diabetes. *Diabetes,* **52,** 536–541.

Ekberg, K., Johansson, B.-L., and Wahren, J. (2001) Stimulation of blood flow by C-peptide in patients with type 1 diabetes. *Diabetologia,* **44(suppl 1),** A323.

Finotti, P., and Palatini, P. (1986) Reduction of erythrocyte ($Na^+$-$K^+$)ATPase activity in type 1 (insulin-dependent) diabetic subjects and its activation by homologous plasma. *Diabetologia,* **26,** 623–628.

Flatt, P. R., Swanston-Flatt, S. K., Hampton, S. M., Bailey, C. J., and Marks, V. (1986) Specific binding of the C-peptide of proinsulin to cultured B-cells from a transplantable rat islet cell tumor. *Biosci. Rep.,* **6,** 193–199.

Forst, T., Dufayet De La Tour, D., Kunt, T., Pfützner, A., Goitom, K., Pohlmann, T., Schneider, S., Johansson, B.-L., Wahren, J., Löbig, M., Engelbach, M., Beyer, J., and Vague, P. (2000) Effects of proinsulin C-peptide on nitric oxide, microvascular blood flow and erythrocyte $Na^+K^+$ATPase activity in diabetes mellitus type 1. *Clin. Sci.*, **98,** 283–290.

Grunberger, G., Qiang, X., Li, Z.-G., Mathews, S., Sbrissa, D., Shisheva, A., and Sima, A. (2001) Molecular basis of the insulinomimetic effects of C-peptide. *Int. J. Exp. Diabetes Res.*, **2,** 156–157.

Hamm, H. E. (1998) The many faces of G protein signaling. *J. Biol. Chem.,* **273,** 669–672.

Hansen, A., Johansson, B.-L., Wahren, J., and Bibra von, H. (2002) C-peptide exerts beneficial effects on myocardial blood flow and function in patients with type 1 diabetes. *Diabetes,* **51,** 3077–3082.

Henriksson, M., Pramanik, A., Shafqat, J., Zhong, Z., Tally, M., Ekberg, K., Wahren, J., Rigler, R., Johansson, J., and Jörnvall, H. (2001) Specific binding of proinsulin C-peptide to detergent-solubilised human skin fibroblasts. *Biochem. Biophys. Res. Commun.,* **280,** 423–427.

Henriksson, M., Shafqat, J., Liepinsh, E., Tally, M., Wahren, J., Jörnvall, H., and Johansson, J. (2000) Unordered structure of proinsulin C-peptide in aqueous solution and in the presence of lipid vesicles. *Cell. Mol. Life Sci.,* **57,** 337–342.

Hoogwerf, B., Bantle, J., Gaenslen, H., Greenberg, B., Senske, B., Francis, R., and Goetz, F. (1986) Infusion of synthetic human C-peptide does not affect plasma glucose, serum insulin, or plasma glucagon in healthy subjects. *Metabolism,* **35,** 122–125.

Huang, D.-Y., Richter, K., Breidenbach, A., and Vallon, V. (2002) Human C-peptide acutely lowers glomerular hyperfiltration and proteinuria in diabetic rats: A dose-response study. *Naunyn Schmiedebergs Arch. Pharmacol.,* **365,** 67–73.

Ido, Y., Vindigni, A., Chang, K., Stramm, L., Chance, R., Heath, W., DiMarchi, R., DiCera, E., and Williamson, J. (1997) Prevention of vascular and neural dysfunction in diabetic rats by C-peptide. *Science,* **277,** 563–566.

Jehle, P., Lutz, M., and Fussgänger, R. (1996) High affinity binding sites for proinsulin in human IM-9 lymphoblasts. *Diabetologia,* **39,** 421–432.

Jehle, P., Fussgänger, R., Angelus, N., Jungwirth, R., Saile, B., and Lutz, M. (1999) Proinsulin stimulates growth of small intestinal crypt-like cells acting via specific receptors. *Am. J. Physiol.,* **276,** E262–E268.

Johansson, B.-L., Borg, K., Fernqvist-Forbes, E., Kernell, A., Odergren, T., and Wahren, J. (2000) Beneficial effects of C-peptide on incipient nephropathy and neuropathy in patients with type I diabetes—a three-month study. *Diabet. Med.*, **17,** 181–189.

Johansson, B.-L., Borg, K., Fernqvist-Forbes, E., Odergren, T., Remahl, S., and Wahren, J. (1996) C-peptide improves autonomic nerve function IDDM patients. *Diabetologia,* **39,** 687–695.

Johansson, B.-L., Linde, B., and Wahren, J. (1992a) Effects of C-peptide on blood flow, capillary diffusion capacity and glucose utilization in the exercising forearm of type I (insulin-dependent) diabetic patients. *Diabetologia,* **35,** 1151–1158.

Johansson, B.-L., Pernow, J., and Wahren, J. (2003) C-peptide increases forearm blood flow in patients with type 1 diabetes via a nitric oxide dependent mechanism. *Am. J. Physiol. Endocrinol. Metab.,* **285,** E864–E870.

Johansson, B.-L., Sjöberg, S., and Wahren, J. (1992b) The influence of human C-peptide on renal function and glucose utilization in type I (insulin-dependent) diabetic patients. *Diabetologia,* **35,** 121–128.

Johansson, B.-L., Sundell, J., Ekberg, K., Jonsson, C., Seppänen, M., Raitakari, O., Luotolahti, M., Nuutila, P., Wahren, J., and Knuuti, J. (2004) C-peptide improves adenosine-induced myocardial vasodilation in type 1 diabetes patients. *Am. J. Physiol. Endocrinol. Metab.,* **286,** E14–E19.

Johansson, J., Ekberg, K., Shafqat, J., Henriksson, M., Chibalin, A., Wahren, J., and Jörnvall, H. (2002) Molecular effects of proinsulin C-peptide. *Biochem. Biophys. Res. Commun.,* **295,** 1035–1040.

Kitamura, T., Kimura, K., Jung, B., Makondo, K., Sakane, N., Yoshida, T., and Saito, M. (2002) Proinsulin C-peptide activates cAMP response element-binding proteins through the p38 mitogen-activated protein kinase pathway in mouse lung capillary endothelial cells. *Biochem. J.,* **366,** 737–744.

Kitamura, T., Kimura, K., Okamoto, S., Canas, X., Sakane, N., Yoshida, T., and Saito, M. (2001) Proinsulin C-peptide rapidly stimulates mitogen-activated protein kinases in Swiss 3T3 fibroblasts: Requirement of protein kinase C, phosphoinositide 3-kinase and pertussis toxin-sensitive G-protein. *Biochem. J.,* **355,** 123–129.

Li, Z.-G., Qiang, X., Sima, A., and Grunberger, G. (2001) C-peptide attenuates protein tyrosine phosphatase activity and enhances glycogen synthesis in L6 myoblasts. *Biochem. Biophys. Res. Commun.,* **280,** 615–619.

Lindström, K., Johansson, C., Johnsson, E., and Haraldsson, B. (1996) Acute effects of C-peptide on the microvasculature of isolated perfused skeletal muscle and kidneys in rat. *Acta Physiol. Scand.,* **156,** 19–25.

Ling, L., Oshida, Y., Kusunoki, M., Yamanouchi, K., Johansson, B.-L., Wahren, J., and Sato, Y. (1999) Rat C-peptide I and II stimulate glucose utilization in STZ-induced diabetic rats. *Diabetologia,* **42,** 958–964.

Maiti, S., Haupts, U., and Webb, W. (1997) Fluorescence correlation spectroscopy: Diagnostics for sparse molecuales. *Proc. Natl. Acad. Sci. U. S. A.,* **94,** 11753–11757.

McMillan, D., Utterback, N., and La Puma, J. (1978) Reduced erythrocyte deformability in diabetes. *Diabetes,* **27,** 895–901.

Mullins, D., Zhang, X., and Hawes, B. (2002) Activation of extracellular signal regulated protein kinase by neuropeptide Y and pancreatic polypeptide in CHO cells expressing the NPY Y(1), Y(2), Y(4) and Y(5) receptor subtypes. *Regul. Peptides,* **105,** 65–73.

Ohtomo, Y., Aperia, A., Sahlgren, B., Johansson, B.-L., and Wahren, J. (1996) C-peptide stimulates rat renal tubular $Na^+$,$K^+$ATPase activity in synergism with neuropeptide Y. *Diabetologia,* **39,** 199–205.

Ohtomo, Y., Bergman, T., Johansson, B.-L., Jörnvall, H., and Wahren, J. (1998) Differential effects of proinsulin C-peptide fragments on $Na^+$,$K^+$-ATPase activity of renal tubule segments. *Diabetologia,* **41,** 287–291.

Pramanik, A., Ekberg, K., Zhong, Z., Shafqat, J., Henriksson, M., Tally, M., Tibell, A., Jansson, O., Wahren, J., Jörnvall, H., Rigler, R., and Johansson, J. (2001) C-peptide binding to human cell membranes: importance of $Glu^{27}$. *Biochem. Biophys. Res. Commun.,* **284,** 94–98.

Rigler, R., Pramanik, A., Jonasson, P., Kratz, G., Jansson, O., Nygren, P.-Å., Ståhl, S., Ekberg, K., Johansson, B.-L., Uhlén, S., Uhlén, M., Jörnvall, H., and Wahren, J. (1999) Specific binding of proinsulin C-peptide to human cell membranes. *Proc. Natl. Acad. Sci. U. S. A.,* **96,** 13318–13323.

Scalia, R., Coyle, K., Levine, B., Booth, G., and Lefer, A. (2000) C-peptide inhibits leukocyte-endothelium interaction in the microcirculation during endothelial dysfunction. *FASEB. J.,* **14,** 2357–2364.

Shafqat, J., Juntti-Berggren, L., Zhong, Z., Ekberg, K., Köhler, M., Berggren, P.-O., Johansson, J., Wahren, J., and Jörnvall, H. (2002) Proinsulin C-peptide and its analogues induce intracellular $Ca^{2+}$ increases in human renal tubular cells. *Cell. Mol. Life Sci.,* **59,** 1185–1189.

Shashkin, P., Jiao, Y., Westerblad, H., and Katz, A. (1997) C-peptide does not alter carbohydrate metabolism in isolated mouse muscle. *Am. J. Physiol.,* **272,** E245–E247.

Sima, A., Zhang, W., Sugimoto, K., Henry, D., Li, Z., Wahren, J., and Grunberger, G. (2001) C-peptide prevents and improves chronic type 1 diabetic neuropathy in the BB/Wor rat. *Diabetologia,* **44,** 889–897.

Tsimaratos, M., Roger, F., Chabardès, D., Mordasini, D., Hasler, U., Doucet, A., Martin, P. Y., and Féraille, E. (2003) C-peptide stimulates Na,K-ATPase via PKC alpha in rat medullary thick ascending limb. *Diabetologia,* **46,** 124–131.

Wahren, J., Ekberg, K., Johansson, J., Henriksson, M., Pramanik, A., Johansson, B.-L., Rigler, R., and Jörnvall, H. (2000) Role of C-peptide in human physiology. *Am. J. Physiol. Endocrinol. Metab.,* **278,** E759–E768.

Wallerath, T., Kunt, T., Forst, T., Closs, E., Lehmann, R., Flohr, T., Gabriel, M., Schäfer, D., Göpfert, A., Pfützner, A., Beyer, J., and Förstermann, U. (2003) Stimulation of endothelial nitric oxide synthase by proinsulin C-peptide. *Nitric Oxide,* **9,** 95–102.

Wójcikowski, C., Maier, V., Dominiak, K., Fussgänger, R., and Pfeiffer, E. (1983) Effects of synthetic rat C-peptide in normal and diabetic rats. *Diabetologia,* **25,** 288–290.

Young, L., Ikeda, Y., Scalia, R., and Lefer, A. (2000) C-peptide exerts cardioprotective effects in myocardial ischemia-reperfusion. *Am. J. Physiol. Heart Circ.,* **279,** H1453–H1459.

Zierath, J., Handberg, A., Tally, M., and Wallberg-Henriksson, H. (1996) C-peptide stimulates glucose transport in isolated human skeletal muscle independent of insulin receptor and tyrosine kinase activation. *Diabetologia,* **39,** 306–313.

